# The Antiviral Effect of the Chemical Compounds Targeting DED/EDh Motifs of the Viral Proteins on Lymphocytic Choriomeningitis Virus and SARS-CoV-2

**DOI:** 10.3390/v13071220

**Published:** 2021-06-24

**Authors:** Mya Myat Ngwe Tun, Kouichi Morita, Takeshi Ishikawa, Shuzo Urata

**Affiliations:** 1 Department of Virology, Institute of Tropical Medicine and Leading Program, Graduate School of Biomedical Science, Nagasaki University, 1-12-4 Sakamoto, Nagasaki 852-8523, Japan; myamyat@tm.nagasaki-u.ac.jp (M.M.N.T.); moritak@nagasaki-u.ac.jp (K.M.); 2 Department of Chemistry, Biotechnology and Chemical Engineering, Graduate School of Science and Engineering, Kagoshima University, 1-21-40 Korimoto, Kagoshima 890-0065, Japan; 3 National Research Center for the Control and Prevention of Infectious Diseases (CCPID), Nagasaki University, 1-12-4 Sakamoto, Nagasaki 852-8523, Japan

**Keywords:** Lymphocytic choriomeningitis virus (LCMV), SARS-CoV-2, antivirals, exonuclease (ExoN) motif, DED/EDh motif

## Abstract

Arenaviruses and coronaviruses include several human pathogenic viruses, such as Lassa virus, Lymphocytic choriomeningitis virus (LCMV), SARS-CoV, MERS-CoV, and SARS-CoV-2. Although these viruses belong to different virus families, they possess a common motif, the DED/EDh motif, known as an exonuclease (ExoN) motif. In this study, proof-of-concept studies, in which the DED/EDh motif in these viral proteins, NP for arenaviruses, and nsp14 for coronaviruses, could be a drug target, were performed. Docking simulation studies between two structurally different chemical compounds, ATA and PV6R, and the DED/EDh motifs in these viral proteins indicated that these compounds target DED/EDh motifs. The concentration which exhibited modest cell toxicity was used with these compounds to treat LCMV and SARS-CoV-2 infections in two different cell lines, A549 and Vero 76 cells. Both ATA and PV6R inhibited the post-entry step of LCMV and SARS-CoV-2 infection. These studies strongly suggest that DED/EDh motifs in these viral proteins could be a drug target to combat two distinct viral families, arenaviruses and coronaviruses.

## 1. Introduction

Several arenaviruses cause hemorrhagic fever in humans. Lassa virus (LASV) is the causative agent of Lassa fever. In addition, evidence indicates that the globally distributed prototypic arenavirus Lymphocytic choriomeningitis virus (LCMV), is a neglected human pathogen of clinical significance in congenital viral infection and transplant recipients’ infection. Current arenavirus antiviral drug therapy is restricted using the nucleoside analog ribavirin (Rib), which is only partially effective, and is associated with significant side effects. Arenavirus possesses a negative-strand RNA as a viral genome and encodes only four viral proteins: a glycoprotein precursor, GPC; an RNA-dependent RNA polymerase (RdRp), L; a matrix protein, Z; and the nucleoprotein, NP. NP is the most abundant and multifunctional protein during viral infection, with key roles in host immunosuppression, viral replication, and encapsidation of the viral genome [1].

Coronaviruses are also a major threat to public health. The most notable infections are severe acute respiratory syndrome (SARS), Middle East respiratory syndrome (MERS), and Coronavirus disease 2019 (COVID-19), caused by SARS-CoV, MERS-CoV, and SARS-CoV-2, respectively. Due to the large outbreak of COVID-19 worldwide, many efforts have been made to develop vaccines and antivirals against COVID-19. However, the appearance of the escape mutant virus against these developed vaccines and antivirals has been a significant concern. Therefore, the development of an effective treatment against COVID-19 is urgently needed. Coronaviruses are enveloped viruses with single-stranded positive-sense RNA genomes of approximately 30 kb. There are approximately 15 predicted open reading frames (ORFs), including ORF1a and ORF1b, spike (S), envelope (E), membrane (M), and nucleocapsid (N). Among these ORFs, nsp14 is known to have bifunctional roles, 3′ to 5′ exoribonuclease (ExoN) and guanine-N7-methyltransferase domains [2].

Arenaviruses NP and coronaviruses nsp14 possess a common motif known as a DED/EDh motif, which has ExoN activity as described above. The role of ExoN activity in the viral life cycle is known to avoid the innate immune response by degrading produced viral RNA, which can be recognized by the RNA sensors. Recent studies have shown the roles of these DED/EDh motifs in viral replication. In this study, proof-of-concept experiments were performed using chemical compounds, which could bind to the DED/EDh motifs in LCMV NP and SARS-CoV-2 nsp14 in silico, respectively, and inhibited the post-entry step of the LCMV and SARS-CoV-2. These results strongly suggest the potential of the DED/EDh motifs as a novel drug target to combat several highly pathogenic viruses belonging to different virus families, including *Arenaviridae* and *Coronaviridae*.

## 2. Materials and Methods

### 2.1. Cells and Compounds

Vero 76, Vero E6, and A549 cells were obtained as described previously [3] and maintained in Dulbecco’s Modified Eagle’s Medium (DMEM) supplemented with 10% fetal bovine serum (FBS) and 1% penicillin/streptomycin. Aurintricarboxylic acid (ATA) and pontacyl violet 6R (PV6R) were obtained from Santa Cruz (sc-3525A) and Tokyo Chemical Industry (P0593-1G), respectively.

### 2.2. Viruses

Tri-segmented recombinant LCMV (3rLCMV/GFP), which expresses green fluorescent protein (GFP) upon infection, was rescued using BHK-21 cells as described previously [4]. The SARS-CoV-2 (JPN/TY/WK-521), which was isolated and provided by the National Institute of Infectious Diseases, Japan [5], was propagated in Vero E6 cells.

### 2.3. Cell Viability Assay

Cell viability of A549 and Vero 76 cells after ATA and PV6R treatment was assessed using the CellTiter-Glo Luminescent Cell Viability Assay (Promega, Madison, WI, USA), which determines the number of viable cells in a culture based on ATP levels. A549 and Vero 76 cells (2 × 10^4^ cells/well) were seeded in 96 well plate to form a monolayer. After cell seeding, the cells were treated with ATA, PV6R, and DMSO as a control. At 24 and 48 h post-treatment, the culture supernatant was removed, and CellTiter-Glo reagent was added. Thereafter, the assay was performed according to the manufacturer’s recommendations using SpectraMAX iD5 (Molecular Device, San Jose, CA, USA). The viability of DMSO-treated control cells was set to 1.0.

### 2.4. Compounds’ Treatments

A549 and Vero 76 cells (2 × 10^4^ cells/well) were seeded in 96 well plates. The cells were infected with 3rLCMV/GFP or SARS-CoV-2. At 1 h post-infection (p.i.), media was replaced with fresh media containing DMSO, ATA, or PV6R. At 24 h p.i. for 3rLCMV/GFP and at 48 h p.i. for the SARS-CoV-2, culture supernatant was collected to measure the viral titer. To visualize the infected cells, the 3rLCMV/GFP infected cells were fixed with 4% paraformaldehyde (PFA) at 20 h p.i.

### 2.5. Titration of 3rLCMV/GFP

Vero 76 cells (2 × 10^4^ cells/well) were seeded one day prior to virus infection in 96 well plates. Culture supernatant upon the compounds’ treatment was collected. Ten times dilution of the virus samples were loaded on top of the Vero 76 cells after removal of the culture medium. At 20 h p.i., cells were fixed with 4% PFA, and the number of GFP-positive cells was counted manually and normalized as fluorescent focus unit (FFU)/mL.

### 2.6. Plaque Assay for SARS-CoV-2

Plaque assays were performed using Vero E6 cell monolayer in a 24-well plate. Briefly, all samples were diluted from 1:10 to 1:10,000 dilutions with a cell culture medium and inoculated on top of the cells in quadruplicate at 200 µL per well. After incubation at 37 °C for 1 h, infected cells were overlaid with 500 µL of 1.25% methylcellulose 4000 in 2% FCS MEM. The plates were then incubated at 37 °C for 4 days. The plates were washed with PBS (-) to remove the methylcellulose, fixed with 4% PFA (Wako, Osaka, Japan) for 30 min at room temperature, rinsed, and stained with crystal violet. Plaques were counted and expressed as plaque forming units (PFU/mL).

### 2.7. Measurement of SARS-CoV-2 RNA in the Cell

Isogen-II (Nippon Gene, Toyama, Japan) was used to isolate and prepare RNA from cells according to the manufacturer’s instruction. A volume of 5 μL of RNA was used for quantitative real-time RT-PCR (qRT-PCR), and amplification of the nucleocapsid (N) gene was performed using a total of 20 µL of reaction mixture, consisting of 5 μL of Taqman master mix, 7 µL of nuclease-free water, 1 µL of 0.5 µM forward and reverse primers, and 1 μL of 0.25 µM probe with SARS-CoV-2 N primers of TaqMan Fast Virus 1-Step Master Mix (Life Technologies, Carlsbad, CA, USA). The primers and probes were described in a previous report [5].

### 2.8. Docking Simulation

The atomic coordinates of the X-ray structure of the LCMV NP were downloaded from the PDB (PDB-ID: 4O6H) [6]. On the other hand, homology modeling was performed to obtain the atomic coordinates of SARS-CoV-2 nsp14 using Modeller10.0 [7] because X-ray structures were not reported. In the homology modeling, the X-ray structure of SARS-CoV nsp14 (PDB-ID: 5C8U) was used as a template [8]. Binding structures of ATA and PV6R to the DED/EDh motif of LCMV and SARS-CoV-2 were predicted by docking simulation using AutoDock Vina [9]. A cubic region of 26.3 × 26.3 × 26.3 Å, whose center was located at the center of mass of the five amino acids of the DED/EDh motif, was selected as a search region. EXHAUSTIVENESS was set to 20. The initial atomic coordinates of ATA and PV6R were downloaded from PubChem (CIDs were 2259 and 112806, respectively).

### 2.9. Statistical Analysis

Excel and GraphPad Prism 5 (GraphPad Software, Inc., San Diego, CA, USA) software were used for all statistical analyses. Quantitative data were presented as the mean ± SD from at least three independent experiments (unless indicated otherwise). For all calculations, *p* < 0.05, was considered significant and was represented using an asterisk (*). Group comparisons were performed using one-way analysis of variance (ANOVA), followed by Dunnett’s multiple comparison test. Welch’s *t*-test was used to compare the two groups.

## 3. Results

### 3.1. Cell Viability against ATA and PV6R Treatment

**Figure 1:**
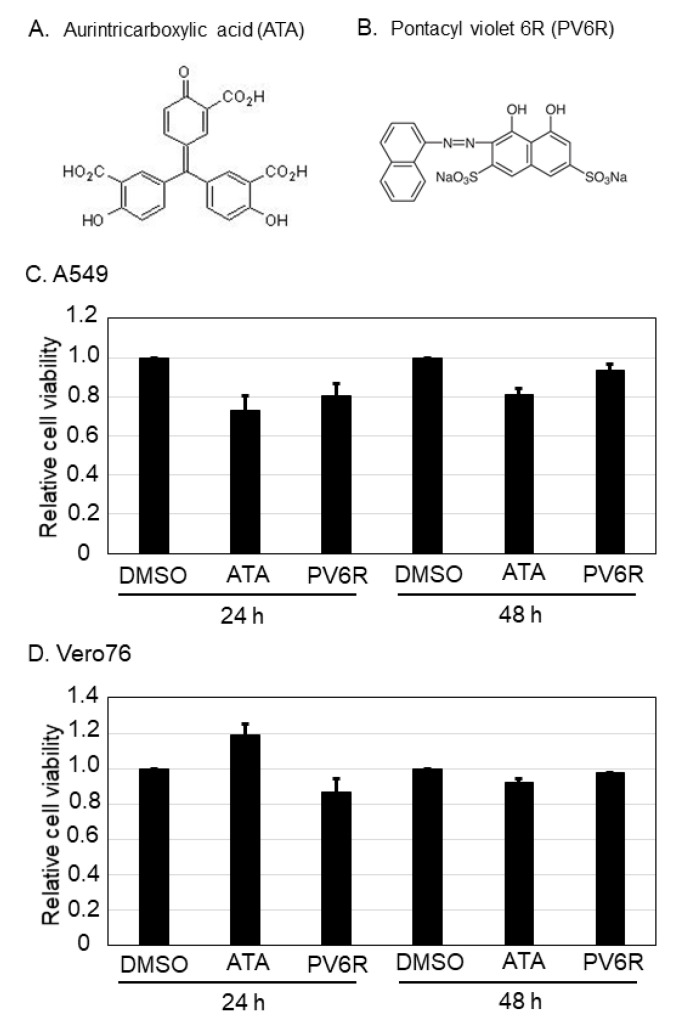
Chemical structure of aurintricarboxylic acid (ATA) and pontacyl violet 6R (PV6R), and their cell viabilities upon the treatments on A549 and Vero 76 cell lines. Chemical structure of ATA (**A**) and PV6R (**B**). Either A549 (**C**) or Vero 76 (**D**) cells were treated with 500 µM of ATA and 200 µM of PV6R for 24 h or 48 h, and their cell viabilities were measured using CellTiter-Glo. Relative cell viability which was normalized with the DMSO control treatment as 1.0 was shown. Data were collected from at least three independent experiments, and provided data correspond to the mean +/− SD.

To examine whether the DED/EDh motifs could be antiviral targets of arenavirus and coronavirus, two chemical compounds (aurintricarboxylic acid [ATA, Figure 1A] and pontacyl violet 6R [PV6R, Figure 1B]), which were previously shown to bind to the DED/EDh motifs and to inhibit ExoN activity [10], were selected. The chemical structures of ATA and PV6R are shown in Figure 1A and 1B, respectively. First, the cell viabilities of two cell lines, A549 (Figure 1C) and Vero 76 (Figure 1D), were examined after ATA and PV6R treatments. Both cell lines were treated with 500 µM (ATA) and 200 µM (PV6R) for 24 and 48 h, and cell viability was measured using CellTiter-Glo as described in the Materials and Methods. In A549 cell lines, cell viability after ATA treatment was 73.4% and 81.7% at 24 and 48 h post-treatment, respectively, compared to the DMSO control treatment. PV6R treatment in A549 cells reduced cell viability to 81.1% and 94.1%, respectively, compared to the DMSO control treatment. In Vero 76 cells, although ATA treatment slightly increased the cell viability (119.7%) at 24 h post-treatment, the same treatment reduced the cell viability to 92.4% at 48 h post-treatment. Cell viabilities in Vero 76 cells upon PV6R treatment were 80.5% and 95.4% at 24 and 48 h post-treatment, respectively.

### 3.2. Both ATA and PV6R Inhibited the Post-Entry Step of the LCMV Infection

**Figure 2:**
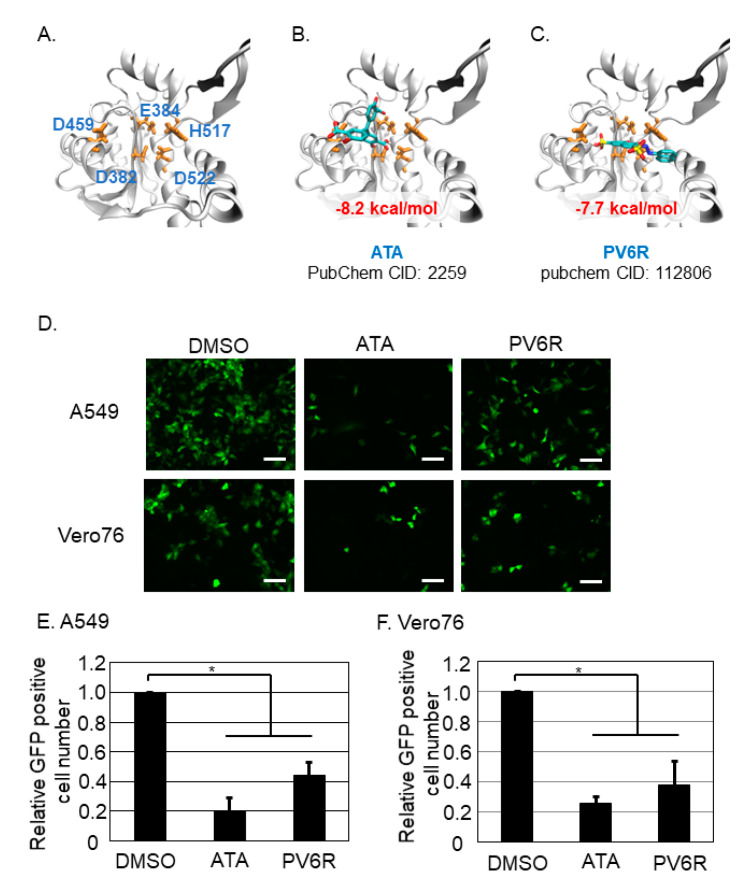
Both ATA and PV6R inhibited the post-entry step of the LCMV infection. (**A**) The structure of the DEDDh motif of LCMV NP. Amino acid number which corresponded to the DEDDh motif was described in BLUE font, together with the amino acid number. (**B**) Docking simulation of the ATA and the DEDDh motif of LCMV NP. (**C**) Docking simulation of the PV6R and the DEDDh motif of LCMV NP. (**D**) Either A549 cells or Vero 76 cells were infected with 3rLCMV/GFP and treated with DMSO, ATA, or PV6R. At 20 h post infection, the cells were fixed and the GFP positive cells were observed with the fluorescent microscope. Bar; 100 µm. GFP positive cell number in A549 (**E**) and in Vero 76 (**F**) were counted and normalized with the GFP positive cell number in DMSO control treatment as 1.0. Data were collected from at least three independent experiments, and shown data correspond to the mean +/− SD (*; *p* < 0.05).

The effect of the DEDDh motif on the structural stability of LCMV NP was examined. The structure of the DEDDh motif of LCMV NP is shown in Figure 2A, and the effects of structural stability upon compound binding were calculated in silico. The calculated binding free energies between the ATA and DEDDh motifs of LCMV NP, and the PV6R and DEDDh motifs of LCMV NP were −8.2 and −7.7 kcal/mol, respectively (Figure 2B,C). With the concentration that did not exhibit apparent cell toxicity, the effects of ATA and PV6R on LCMV infection were examined. To assess this point, tri-segment recombinant LCMV expressing GFP upon infection (3rLCMV/GFP) was used [4]. GFP expression was reflected in the replication and transcription of LCMV in the cell. A549 cells were infected with 3rLCMV/GFP at an MOI of 0.1, and Vero 76 was infected with 3rLCMV/GFP at an MOI of 0.01. At 1.5 h post-infection (p.i.), the culture media was replaced with fresh media containing DMSO, ATA, or PV6R. Cells were fixed with 4% paraformaldehyde (PFA) at 20 h p.i., captured using a fluorescent microscope (Figure 2D), and GFP-positive cells were counted (Figure 2E,F). Treatment with ATA reduced the number of GFP-positive cells to 20.1% and 25.2% compared to DMSO treatment in A549 and Vero 76 cells, respectively. Treatment with PV6R reduced the number of GFP-positive cells to 44.2% and 37.8% compared to DMSO treatment in A549 and Vero 76 cells, respectively.

### 3.3. ATA, but Not PV6R, Inhibited the Propagation of the LCMV

**Figure 3:**
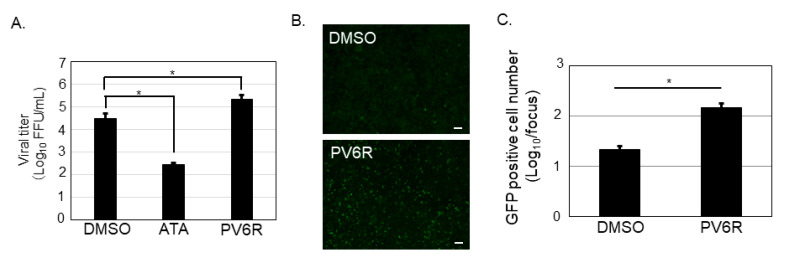
ATA, but not PV6R, inhibited LCMV production. (**A**) Culture supernatant of the Vero 76 cells infected with 3rLCMV/GFP and treated with DMSO, ATA (500 µM), or PV6R (200 µM), were used to measure the viral titer. (**B**,**C**) 3rLCMV/GFP and Vero 76 cells were pre-treated with PV6R (200 µM) and allowed for the infection. PV6R was not included in the culture media after the infection. At 20 h post infection, the cells were fixed and the GFP positive cells were observed with the fluorescent microscope. Bar; 200 µM (**B**). Counted GFP positive cells were plotted from both DMSO and PV6R treatments (**C**). Data were collected from at least three independent experiments, and shown data correspond to the mean +/− SD (* *p* < 0.05).

Because a significant reduction in the number of GFP-positive cells was observed in ATA and PV6R treatments in A549 and Vero 76 cells, the viral production from the ATA and PV6R treated Vero 76 cells was also evaluated. Vero 76 cells were infected with 3rLCMV/GFP for 1.5 h, and the culture medium was replaced with fresh media containing DMSO, ATA (500 µM), or PV6R (200 µM). At 24 h p.i., the culture supernatant was collected to measure their viral titer using Vero 76 cells. ATA treatment resulted in an approximately 100-fold reduction in the viral titer, compared to DMSO treatment. In contrast, PV6R treatment increased the viral titer by approximately 10 times, compared to the DMSO control treatment (Figure 3A). To observe if PV6R affects steps other than the replication step in the cells of the LCMV replication, PV6R was incubated with both 3rLCMV/GFP and Vero 76 cells, respectively, before the infection. After 1 h incubation, the virus with PV6R and Vero 76 cells with PV6R were mixed together, allowing the infection. At 1.5 h p.i., infected cells were washed and kept in incubation without any compounds. Infected cells were fixed at 20 h p.i. and observed under the fluorescent microscope (Figure 3B). PV6R treatment significantly increased the GFP positive cell number (6.7 times) compared to that of the DMSO treatment (Figure 3C).

### 3.4. Both ATA and PV6R Inhibited SARS-CoV-2 Replication and Propagation

**Figure 4:**
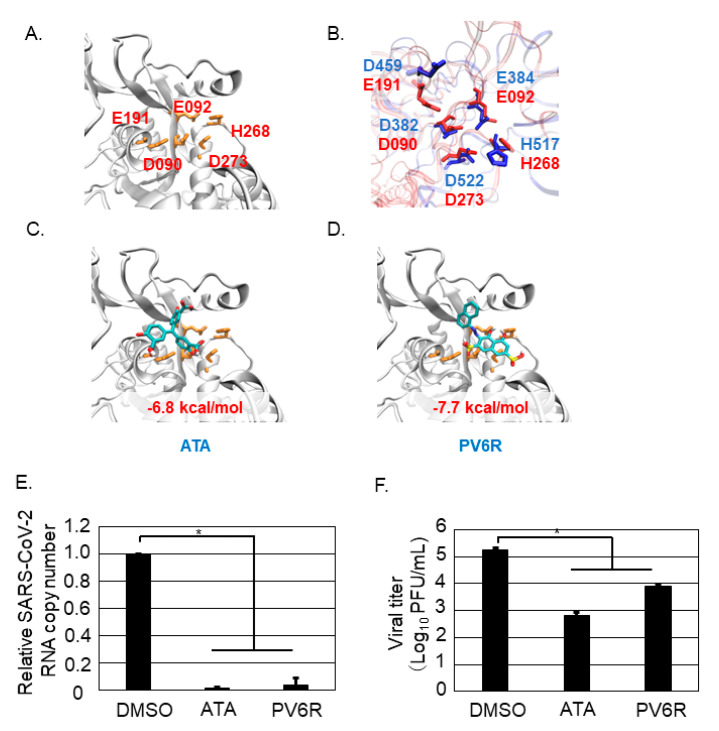
Both ATA and PV6R inhibited SARS-CoV-2 infection and propagation. (**A**) Homology modeling of the DEEDh motif of the SARS-CoV-2 nsp14. Amino acid number which corresponded to the DEEDh motif was described in RED font together with the amino acid number. (**B**) Overlap of the SARS-CoV-2 nsp14 and LCMV NP DED/EDh motifs was shown. Amino acids of the DED/EDh motifs in SARS-CoV-2 nsp14 and LCMV NP were colored in RED and BLUE, respectively. (**C**) Docking simulation of the ATA and the homology model of the SARS-CoV-2 nsp14. (**D**) Docking simulation of the PV6R and the homology model of the SARS-CoV-2 nsp14. Vero 76 cells were infected with SARS-CoV-2 and treated with DMSO, ATA, or PV6R, respectively. The viral copy number in the cells at 24 h p.i. was measured. Relative viral copy number normalized with the DMSO control treatment in Vero 76 cells was shown (**E**). Vero 76 cells were infected with SARS-CoV-2 and treated with DMSO, ATA, or PV6R, respectively, and the viral titer was measured at 48 h p.i. (**F**). Data were collected from at least three independent experiments, and shown data correspond to the mean +/− SD (* *p* < 0.05).

Coronaviruses’ nsp14 possess the DEEDh motif (equivalent to the DEDDh motif), which contributes to anti-interferon (IFN) activity and virus replication [11]. The crystal structure of the nsp14 of SARS-CoV-2 has not been reported; thus, homology modeling was performed to predict SARS-CoV-2 nsp14 structure using SARS-CoV nsp14 (Figure 4A) [8]. The predicted homology model of SARS-CoV-2 nsp14 overlapped with the DEDDh motif of LCMV NP (Figure 4B). Furthermore, docking simulation between SARS-CoV-2 nsp14 and the compounds (ATA and PV6R) was also performed (Figure 4C,D). For both compounds, the binding structures that strongly interacted with the 5 amino acids of the DEEDh motif were obtained. The calculated binding free energies were −6.8 (ATA) and −7.7 (PV6R) kcal/mol, respectively. To examine if ATA and PV6R inhibited the SARS-CoV-2 in vitro, Vero76 cells were infected with SARS-CoV-2, and at 1.5 h p.i., the culture media was replaced with fresh media containing DMSO, ATA (500 µM), or PV6R (200 µM). At 24 h p.i., RNA was collected from the infected cells to quantify the viral RNA produced from the SARS-CoV-2 infection (Figure 4E). ATA and PV6R treatments reduced 66.6-fold and 25-fold of the viral genome copy number compared to the DMSO treatment, respectively (Figure 4E). Furthermore, to examine if these compounds inhibited the propagation of the SARS-CoV-2, virus infection was proceeded in the same way as the above experiment and the culture supernatant was collected to evaluate the produced infectious virion in the culture supernatant. In Vero 76 cells, ATA and PV6R treatments reduced the production of the infectious virion to 0.35% and 4.2%, respectively, compared to the DMSO control treatment.

## 4. Discussion

Arenaviruses and coronaviruses are important human pathogens. LASV is the major pathogen of Lassa fever and belongs to the *Areanaviridae* family. LCMV also belongs to *Arenaviridae* and is harmful to pregnant women and transplant recipients [1]. SARS-CoV, MERS-CoV, and SARS-CoV-2 are other important human pathogens that belong to *Coronaviridae*. Although these two virus families (*Arenaviridae* and *Coronaviridae*) have different features, arenavirus possesses single-stranded negative-sense RNA and coronavirus possesses single-stranded positive-sense RNA as viral genomes, arenavirus NP and coronavirus nsp14 encode DED/EDh motifs, which have been shown to play important roles in antagonizing the innate immune response (also known as an ExoN motif) and in viral genome replication.

The first discovery of ExoN activity in human pathogenic viruses was from the coronavirus. The DEEDh motif of nsp14 as an ExoN was shown by biochemical experiments [12]. It is now widely accepted that the ExoN of nsp14 is conserved among coronaviruses [13]. In addition, the DEEDh motif of nsp14 is important for viral replication apart from the anti-IFN function [11]. Determining the structure of the SARS-CoV nsp14-nsp10 complex also provided evidence of its ExoN activity [8]. Accordingly, the ExoN activity of coronavirus nsp14 appears to be an important drug target to combat coronaviruses, including SARS-CoV-2 [14]. The structure of SARS-CoV-2 nsp14 has not yet been published in the protein data bank [15]; therefore, a predicted structure based on the SARS-CoV nsp14 was constructed (Figure 4A). Using this model, we calculated the stability of the DEEDh motif of SARS-CoV-2 nsp14 upon binding two chemical compounds, ATA and PV6R, in silico. During this study, a similar attempt was reported, and the interaction between PV6R and SARS-CoV-2 nsp14 was examined in silico as well [16]. Our study is the first to reveal that both ATA and PV6R inhibited SARS-CoV-2 replication and propagation in vitro (Figure 4E,F), as predicted from the in silico studies.

In the case of arenaviruses, a study was performed to identify specific amino acids (D382 and G385) in LCMV NP, which corresponded to antagonizing the IFN activity and was involved in genome replication using a reverse genetics system and the mini-genome system [17]. It was demonstrated that the point mutations in D382 and G385 in LCMV NP did not affect genome replication; however, it affected the anti-IFN activity. In contrast, deletion of these amino acids (D382 and G385) abolished the genome replication and an anti-IFN activity. These results suggest that the overall structure of the DEDDh motif in NP could affect not only anti-IFN activity, but also genome replication. A similar approach was used with LASV to show the same motif on anti-IFN activity in NP (D389 and G392) [18]. The authors of this article described that LASV NP-G392A recombinant virus was not rescued, although NP-D389A/G392A and NP-D389T/G392A double mutants were rescued. These results suggest that disruption of the DEDDh motif in NP could affect genome replication. Upon determination of the LASV and LCMV NP structures, it became apparent that the DEDDh motif in arenavirus NP corresponds to coronavirus nsp14 as an ExoN motif [6,19,20,21].

Based on the increasing evidence that the DED/EDh motifs of arenavirus NP and coronavirus nsp14 could be a target to inhibit virus replication and propagation, this study attempted in vitro analysis to assess this point as a proof-of-concept study. Although we did not experimentally show that ATA and PV6R bind to the arenavirus NP and coronavirus nsp14 in this study, we reported the antiviral effects of ATA and PV6R, which were previously shown to inhibit ExoN activity in biochemical studies [10], on the post-entry step of LCMV and SARS-CoV-2. Due to our experimental design, we could not precisely point out the compounds’ target of the post-entry step in the viral life cycle. However, our in silico study showed that both compounds exhibited a strong potential to bind to the DEDDh motif of LCMV NP and accordingly inhibited the post-entry step of LCMV (Figure 2E,F). Furthermore, ATA and PV6R exhibited a strong potential to bind to the DEEDh motif of SARS-CoV-2 nsp14 (Figure 4C,D) and affected protein stability. Accordingly, these compounds also inhibited the post-entry step (Figure 4E) and propagation (Figure 4F) of SARS-CoV-2. These results supported the idea that both ATA and PV6R inhibited the arenavirus and coronavirus replication through binding ExoN motif in each viral proteins.

It is worth noting that ATA affects cellular events other than ExoN activity. ATA has been reported to inhibit ZIKV [22], vaccinia virus [23], hepatitis C virus [24], and influenza A and B viruses [25], all of which do not possess the DED/EDh motif. The potential of the ATA to bind SARS-CoV proteins other than the nsp14 [26,27] has also been reported, suggesting that the antiviral effect we observed was not purely due to the disruption of the DEEDh motif of SARS-CoV-2 nsp14. However, the antiviral effect of PV6R against SARS-CoV-2 supports the idea that the DEEDh motif of SARS-CoV-2 nsp14 could be an antiviral target. Although the concentration of the compounds used in this study showed a modest effect on cell viability (Figure 1C,D), in which the newly synthesized ATP was measured, these chemical compounds could still affect the host factors. The 3′-to-5′ ExoN plays major roles in prokaryotic and eukaryotic cells, and among these exonucleases, the DEDDh superfamily is involved in 3′ maturation, nuclear mRNA surveillance, and mRNA decay [28]. Indeed, ATA is known to inhibit nucleases [29], nucleic acid processing enzymes [30], and phosphorylation of extracellular signal-regulated kinase 1/2 [23]. The results from the PV6R, whose chemical structure is different from that of ATA, supported the notion that DED/EDh motifs of arenavirus NP and coronavirus nsp14 could be antiviral targets. The chemical information of PV6R is limited; therefore, it is difficult to discuss how PV6R affects cellular events. The results obtained from our study indicated that PV6R could facilitate the entry process of LCMV infection (Figure 3B,C). Further experiments are required to explore the precise mechanism how PV6R facilitate the LCMV entry process.

In conclusion, this study proved that the DED/EDh motif of two different virus families, arenavirus and coronavirus, could be a drug target. Although ExoNs are also expressed in the host, targeting slight differences in these ExoNs between the host and viral ExoNs, such as the existence of the zinc finger motif, could lead to develop a specific drug [31]. In addition, treating these viral infections with dual and/or triple drugs could increase the antiviral effect and reduce the possible appearance of drug-resistant viruses.

## Data Availability

Not applicable.
